# Anticancer effect of zinc oxide nanoparticles prepared by varying entry time of ion carriers against A431 skin cancer cells *in vitro*

**DOI:** 10.3389/fchem.2022.1069450

**Published:** 2022-12-01

**Authors:** Albandri Yousef Aljohar, Ghazala Muteeb, Qamar Zia, Sahabjada Siddiqui, Mohammad Aatif, Mohd Farhan, Mohd. Farhan Khan, Abdulrahman Alsultan, Azfar Jamal, Adil Alshoaibi, Ejaz Ahmad, Mir Waqas Alam, Md Arshad, Mohd Imran Ahamed

**Affiliations:** ^1^ Department of Clinical Nutrition, College of Applied Medical Science, King Faisal University, Al Ahsa, Saudi Arabia; ^2^ Department of Nursing, College of Applied Medical Science, King Faisal University, Al Ahsa, Saudi Arabia; ^3^ Department of Medical Laboratory Sciences, College of Applied Medical Sciences, Majmaah University, Al Majma'ah, Saudi Arabia; ^4^ Health and Basic Sciences Research Center, Majmaah University, Al Majma'ah, Saudi Arabia; ^5^ Department of Biotechnology, Era’s Lucknow Medical College & Hospital, Era University, Lucknow, India; ^6^ Department of Public Health, College of Applied Medical Science, King Faisal University, Al Ahsa, Saudi Arabia; ^7^ Department of Basic Sciences, King Faisal University, Al Ahsa, Saudi Arabia; ^8^ Faculty of Science, Gagan College of Management & Technology, Aligarh, India; ^9^ Department of Biomedical Sciences, College of Medicine, King Faisal University, Al Ahsa, Saudi Arabia; ^10^ Department of Biology, College of Science, Majmaah University, Al Majma'ah, Saudi Arabia; ^11^ Department of Physics, College of Science, King Faisal University, Al Ahsa, Saudi Arabia; ^12^ Interdisciplinary Biotechnology Unit, Aligarh Muslim University, Aligarh, India; ^13^ Molecular Endocrinology Laboratory, Zoology Department, Lucknow University, Lucknow, India; ^14^ Department of Zoology, Aligarh Muslim University, Aligarh, India; ^15^ Department of Chemistry, Faculty of Science, Aligarh Muslim University, Aligarh, India

**Keywords:** zinc oxide nanoparticles, sol-gel synthesis, malignant cell lines, MTT assay, reactive oxygen species

## Abstract

Although, zinc oxide nanoparticles (ZRTs) as an anti-cancer agent have been the subject of numerous studies, none of the reports has investigated the impact of the reaction entry time of ion-carriers on the preparation of ZRTs. Therefore, we synthesized variants of ZRTs by extending the entry time of NaOH (that acts as a carrier of hydroxyl ions) in the reaction mixture. The anti-proliferative action, morphological changes, reactive oxygen species (ROS) production, and nuclear apoptosis of ZRTs on human A431 skin carcinoma cells were observed. The samples revealed crystallinity and purity by X-ray diffraction (XRD). Scanning electron microscopy (SEM) images of ZRT-1 (5 min ion carrier entry) and ZRT-2 (10 min ion carrier entry) revealed microtubule like morphology. On prolonging the entry time for ion carrier (NaOH) introduction in the reaction mixture, a relative ascent in the aspect ratio was seen. The typical ZnO band with a slight shift in the absorption maxima was evident with UV-visible spectroscopy. Both ZRT-1 and ZRT-2 exhibited non-toxic behavior as evident by RBC lysis assay. Additionally, ZRT-2 showed better anti-cancer potential against A431 cells as seen by MTT assay, ROS generation and chromatin condensation analyses. At 25 μM of ZRT-2, 5.56% cells were viable in MTT test, ROS production was enhanced to 166.71%, while 33.0% of apoptotic cells were observed. The IC_50_ for ZRT-2 was slightly lower (6 μM) than that for ZRT-1 (8 μM) against A431 cells. In conclusion, this paper presents a modest, economical procedure to generate ZRT nano-structures exhibiting strong cytotoxicity against the A431 cell line, indicating that ZRTs may have application in combating cancer.

## 1 Introduction

Nanotechnology is the science of fabrication, characterization and application of particles with nanoscale (1–100 nm) dimensions (Sirelkhatim et al., 2015). Elements with atomic scale possess increased surface area-to-volume ratio than bulk materials (Khan et al., 2019). This nano size range drastically changes their physical, chemical and biological characteristics, and confers different phenomena and functions (Jiang et al., 2018). With these properties of nanomaterials, nanotechnology has now evolved as a cutting-edge technology with several applications in optics, electronics, agribusiness, cosmetology, forensics, biomedical sector and many more (Harun et al., 2017; Siddiqi et al., 2018).

Zinc is a naturally occurring micro-element found essentially in all living organisms (Jiang et al., 2018). Zinc functions as a cofactor for numerous enzymes involved in metabolism, hematopoiesis, and neurobiology (Kambe et al., 2015). The oxide form of zinc (ZnO, Zn^2+^) is chemically stable, biocompatible, less hazardous to the human body (Sirelkhatim et al., 2015; Mandal et al., 2022) exhibiting negligible hemolysis against human red blood cells (Abbasi et al., 2019). It has been demonstrated that ZnO-based materials are biodegradable both in their bulk and nanoparticulate form (Kielbik et al., 2017). Moreover, absorption of nano-form of zinc is high due to its small unit dimension (Jiang et al., 2018). Nano-ZnO is frequently used as an additive in numerous materials and products including ceramics, glass, cement, rubber (e.g., car tyres), pigments, foods (source of Zn nutrient) (Sabir et al., 2014).

Zinc oxide nanoparticles (referred here as ZRTs) possess unique physical and chemical assets, due to its high electron mobility, wide band-gap and elevated exciton energy (Ruszkiewicz et al., 2017). These nanoparticles continue to be among the most widely accepted in a plethora of fields (Akintelu & Folorunso 2020; Berehu et al., 2021). Due to its great biocompatibility, nanoparticulate ZRTs has been designated as a “GRAS” (generally regarded as safe) substance (21 CFR 182.8991) by the US Food and Drug Administration (FDA) (Khan et al., 2006). ZRTs are inexpensive, less hazardous and better biocompatible than other metal oxide nanoparticles. They have been utilized in a variety of medical implications such as anti-microbial, anti-diabetic, anti-inflammatory, anti-aging agent, as well as in healing process and bioimaging (Xiong 2013; Zhang and Xiong, 2015; Kim et al., 2017; Mishra et al., 2017). Due to their special characteristics, ZRTs can be employed therapeutically as anticancer agents (Wiesmann et al., 2020). Cytotoxic activity of ZRTs has been reported against several cancers, including triple-negative breast cancer cells (Stepankova et al., 2021), MCF7 breast cancer cells (Motazedi, et al., 2020), lung adenocarcinoma (Bai et al., 2017), bladder cancer (Zhang et al., 2020), oral cancer (Wang et al., 2018) and liver cancer cells (Rahimi Kalateh Shah Mohammad et al., 2019) as well as chronic myeloid leukemia (Alsagaby et al., 2020).

Globally, the frequency of developing skin cancer has increased due to prolonged exposure to radiation, environmental variations, as well as personal reasons (Zaar et al., 2016; Veisani et al., 2017). Skin cancers consist of cutaneous melanoma (CM) and non-melanoma skin cancer (NMSC). Epidermoid carcinoma of the skin is a non-melanoma malignant tumor of epidermal keratinocytes. Since accelerated growth in epidermis may be associated with skin cancer, the human skin epidermal squamous carcinoma cell line, A431 has emerged as an effective candidate for assessing the anti-cancer properties of different formulations. Several nanostructures like silica (Ahamed, 2013), titanium (Shukla et al., 2011), nickel (Alarifi et al., 2014), gold (Rajendran et al., 2021), and silver (Saber et al., 2018) nanoparticles have reported cytotoxic activity against A431 cell line. However, there are limited studies on bio-activity of chemically synthesized ZRTs on A431 cells. Moreover, to the best of our knowledge, none of the studies evaluated the effect of time of addition of NaOH in the reaction mixture on the synthesis of ZRTs and its anticancer abilities.

In our previous study, we have explored the antineoplastic activity of ZRTs prepared by varying concentrations of sodium hydroxide, NaOH, that act as a carrier of hydroxyl ions (OH^−^) (Khan et al., 2021). In this study, we synthesized ZRTs by means of sol-gel method using cetyl trimethyl ammonium bromide (CTAB) as capping agent and sodium hydroxide (NaOH) as a reducing agent as well as ion carrier, while zinc acetate dihydrate (ZAD) behaves as a precursor for the formation of ZRTs. Variation in the entry time of the ion carrier, NaOH resulted in two different structures, ZRT-1 and ZRT-2. Microscopic and spectroscopic investigations including scanning electron microscopy (SEM), UV-visible spectroscopy, and X-ray diffraction (XRD) confirmed the formation of ZRTs. Further, we evaluated the cytotoxic activities of ZRTs prepared by varying entry time of ion carrier against skin cancer cell line, A431. This approach is simple, affordable and does not require sophisticated instrumentation.

## 2 Materials and methods

### 2.1 Materials

Chemicals including ZAD, NaOH, and the capping agent, CTAB (C_19_H_42_NBr), were procured from E. Merck Ltd. (Mumbai, India) and LobaChemie (Mumbai, India) and utilized as such with no additional purification. Before every test, glasswares, purchased from Borosil, India, were cleaned and sterilized. Double-distilled (DD) water was utilized for the reaction process. Sigma-Aldrich (US) and Hi-Media (India) supplied the chemicals, cell culture media, and supplements used in the cell culture analysis.

### 2.2 Synthesis of ZRTs

The research was carried out using DD water with two separate reactant settings. DD water (180 ml) and 0.36445 g of CTAB were added at room temperature in synthesis - A while magnetic stirring was carefully controlled. This was followed by the addition of 10 ml of 0.1 M solution of ZAD (0.001 mol) and the mixture was kept under stirring for 5 min. To finish, 10 ml of 0.1 M NaOH was added drop-wise to the reaction system after 5 min. The arrangement was kept as such for about half an hour, until a white cloudy substance appeared. The formulation was then washed with DD water and absolute alcohol to remove impurities and to obtain pure form of nanoparticles. The suspension was put into an open petri plate to dry, after which the residual dried material was weighed and measured. The material was stored at −20°C until characterization was done. In synthesis - B, identical steps were performed in formulation of another specimen except for a change in the reaction time for addition of the ion-carriers i.e., NaOH which was increased to 10 min. The synthesized item was stored at −20°C until further use. For the sake of simplicity and to distinguish the zinc oxide nanostructures discussed in previous studies, we will designate them as ZRT-1 (prepared by reaction entry of ion carrier after 5 min) and ZRT-2 (prepared by reaction entry of ion carrier after 10 min). A previously published method was employed to measure the ZRT concentration in a suspension of liquid (McGuffie et al., 2016). The molar concentration of ZRTs suspension was calculated to be around 0.000328 M (328 µM). Depending upon the requirement; the stock solution (328 µM) was diluted to prepare desired ZRT concentration.

### 2.3 Characterization of ZRTs

Utilizing a Bruker D8 ADVANCE (Germany) X-ray diffractometer and an X-ray beam with Cu-K_α_ radiation of a wavelength (λ) equal to 1.54178 Å, a step dimension of 0.01°, and a scanning speed of 0.02 steps/second, the XRD of generated ZRTs were examined. The power generation was set at 40 kV and 40 mA. The Debye-Scherer equation [D = (K.λ)/(d.cos θ)] was used to determine the nano-particulate dimensions exploiting spectral peaks: where, D is crystallite size, k is proportionality constant with no dimensions and a value that is almost unity, λ is X-ray wavelength of Cu-K_α_ radiation (1.54178 Å), θ is full width at half maximum (FWHM) of XRD peaks and is Bragg’s angle. For the 2θ horizontal axis, the position of the diffraction peak pattern on the horizontal plane is θ; the 2θ values are evenly divided to get θ positions (Epp, 2016). The integrated software, Diffracplus, considerably streamlined the calculation. The twin beam PERKIN-ELMER (US) spectrophotometer was used to conduct UV-Visible absorption spectroscopy. The background adjustment was performed using DD water as a reference. With the use of SEM (JEOL JSM-6510 LV, Japan) morphological features (shape and D/L values) of as-synthesized nanostructures were studied.

### 2.4 ZRT-induced red blood cells (RBCs) hemolysis

To determine the amount of hemolysis, a known hematocrit of red blood cells (RBCs) (about 2 × 10^8^ cells/mL) were incubated for 24 h with 1 ml of ZRTs at various concentrations (1, 5, 10, 50, 100, and 200 µg/ml) in a final volume of 2 ml at 37°C. After desired incubation, the reaction mixture was centrifuged at 1,200 g, and the supernatant was collected. The absorbance was measured at 576 nm for released hemoglobin. As a positive control for 100% cell lysis, Triton X-100 (a nonionic surfactant) at a concentration of 0.1% was applied. The result was calculated using the following equation and represented visually as a percentage of 100% cell lysis (Zia et al., 2015):

$$\%\;RBC\;lysis=\left(\frac{{Abs}_{T}-{Abs}_{C}}{{Abs}_{100\%}-{Abs}_{C}}\right)\times 100$$

where AbsT is the absorbance of the supernatant from samples incubated with the drugs, AbsC is the absorbance of the supernatant from the control (PBS), and Abs100% is absorbance in the presence of 0.1% Triton X-100. The results are the mean of three independent experiments.

### 2.5 Cell lines and culture

Human epidermoid carcinoma A431 cell line and kidney epithelial Vero cell line were purchased from the National Center for Cell Sciences (NCCS), Pune, India. The cell lines were subcultured in Dulbecco’s Modified Eagle Medium (DMEM)-F12 medium, which also contained 10% (v/v) fetal calf serum (FCS), sodium bicarbonate (NaHCO_3_) (1.5 g/L), and L-glutamine (2 mM).

### 2.6 Cell viability assay

The MTT [(3-(4,5-dimethylthiazol-2-yl)-2,5-diphenyltetrazolium bromide) tetrazolium] assay (an enzymatic reduction of MTT dye) was used to evaluate the anti-proliferative activity of ZRTs (ZRT-1 and ZRT-2) as per previous study (Khan et al., 2022). A 96-well culture plate with 100 µL of DMEM-F12 was seeded with about 1 × 10^4^ cells and incubated in a CO_2_ incubator overnight. To achieve the appropriate concentrations of 5, 10, and 25 µM, a stock suspension of ZRTs was prepared in DD water and diluted in DMEM-F12 media. After that, cells were treated in triplicate with various ZRT doses and incubated for 24 h. As a control, cells were subjected to media alone. Afterwards, 10 μL MTT was added to cells from a stock solution (in 5 mg/ml phosphate-buffered saline, PBS, pH 7.4) and plate was incubated for specified time till color development. Next, 100 μL DMSO solution was added in each well to solubilize blue crystals and percent viability was computed as described previously (Khan et al., 2021):

$$\%\;Cell\;viability=\left(\frac{{OD}_{treated}}{{OD}_{control}}\right)\times 100$$

### 2.7 Reactive oxygen species generation

ROS intensity was measured in A431-treated cells following published report (Jafri et al., 2019). Cells (1 × 10^4^/well) were cultured overnight in a 96-well cell-culture plate before being subjected to various concentrations of ZRTs for 12 h. After stipulated time period, the cells were mixed with 10 μM of Dichloro-dihydro-fluorescein diacetate (DCFH-DA) dye for 30 min in dark. PBS solution was added twice to replenish the reaction mixture. Images of the intracellular fluorescence intensity were captured using an inverted fluorescence microscope (Zeiss Axio Observer Microscope, Germany). Cells were treated for 12 h on a 96-well black bottom culture plate for the quantitative evaluation of fluorescence intensity. Cells were then stained with DCFH-DA dye for 30 min. Cells were washed with PBS solution (200 µL) to remove unwanted stain. Using a multiwell microplate reader, the fluorescence intensity of ROS production was measured at excitation wavelength of 485 nm with emission wavelength set at 528 nm (Omega Fluostar). Values were expressed as a percentage of the intensity of the fluorescence in comparison to the controls.

### 2.8 Fluorescent nuclear staining

The apoptotic effect of ZRTs was measured using the fluorescent nuclear dye DAPI (4′,6-diamidino-2-phenylindole) (Jafri et al., 2019). ZRTs were applied to A431 cells for 24 h in a 48-well plate. Cells were fixed with 4% paraformaldehyde for 10 min and permeabilized using permeabilizing solution containing 0.5% Triton X-100 reagent and 3% paraformaldehyde. Then, using a fluorescence microscope, cells were stained with DAPI dye (Nikon ECLIPSE Ti-S, Japan).

### 2.9 Statistical analysis

The results are presented as mean ± SD of three independent experiments (*n* = 3). One-way ANOVA and Dunnett’s Multiple Comparison Test was employed for testing the significance of the data using Graph Pad Prism (Version 5.01) software. A *p*-value of ≤0.05 was considered significant.

## 3 Results and discussion

Various physico-chemical methods (Krol et al., 2017; Jin and Jin, 2019); such as sol-gel (Khan et al., 2014, 2016, 2020), solution precipitation (Thein et al., 2015), electrochemical synthesis (Chandrappa and Venkatesha 2012), co-precipitation (Adam et al., 2018), hydrothermal precipitation (Li et al., 2017), sonochemical (Noman and Petrů 2020), mechanochemical (Otis et al., 2021) microemulsions (Han et al., 2016), thermal evaporation (Stanković et al., 2013), spray pyrolysis (Ebin et al., 2012) and microwave-assisted methods (Shinde et al., 2014; Wojnarowicz et al., 2020) have been implemented for the preparation of ZRTs resulting in a wide range of shapes and sizes of NPs. Our previous approaches used for synthesis of ZRTs involved variation in temperatures and stirring speeds, while avoiding sophisticated equipment. In one study, it was demonstrated how ZRTs prepared at two distinct incubation temperature might develop in a variety of ways. These NPs presented the shape of nanoflowers with varied length to diameter ratios (L/D; aspect ratios) (Khan et al., 2014). At ambient temperature, our group also observed the impact of mechanical agitation on ZRTs formation that featured thorn-resembling designs (Khan et al., 2016). Additionally, NPs synthesized at temperatures closer to room temperature exhibited more effectiveness against microorganisms (Khan et al., 2014). Furthermore, neither exorbitant raw materials nor complex machinery are required. These results encouraged us to evaluate the performance of ZnO nanoparticles generated by delaying the entry time of ion carriers (ZRTs) during synthesis. This chemical procedure carried out at close to room temperature is very efficient, economical and is easily mass scalable. We further studied the impact of change in entry time of ion-carriers on ROS production, nuclear condensation and apoptosis in human epidermoid carcinoma A431 cell line.

Previously, we have observed that NaOH containing the hydroxide ions (OH^−^) makes contact with the Zn^2+^ ions and leads to the formation of nano-sized ZnO materials. Thus, during the process of the synthesis of ZRTs, NaOH serves as a carrier of hydroxide ions (OH^−^) (Khan et al., 2020). Adding NaOH to ZAD leads to the formation of hexagonal arrays as zincate ions slowly convert into hydroxyl ions and zinc oxide. This happens as a result of the ZnO crystal acting as a polar crystal and progressively forming its crystal structure from OH^−^ ions. The ZnO nucleus expands and forms ZnO strands when the particles are saturated. The initial structure arises when freshly made strands are deposited on top of preexisting crystallites, which later coalesces into a variety of different microtubule-like structures (Kumar et al., 2013). The CTAB acts as a capping agent that stabilizes the nanostructures.

### 3.1 Characterization of ZRTs

#### 3.1.1 XRD analysis of ZRTs

**FIGURE 1:**
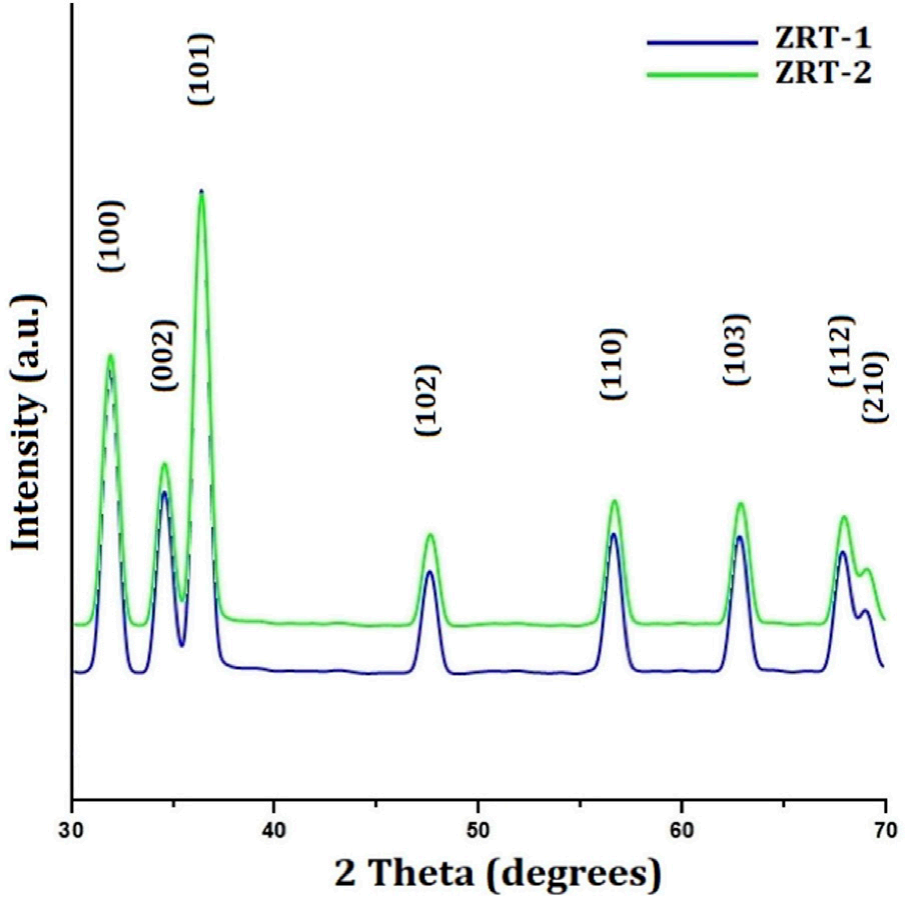
XRD characterization of ZRT samples prepared by varying reaction time of ion-carriers on the synthesis of zinc oxide nanoparticles: ZRT-1 and ZRT-2.

The hexagonal peculiarities of samples of synthesized ZRTs were demonstrated by XRD technique (Figure 1). The X-ray diffraction (XRD) patterns for ZRT-1 and ZRT-2 were provided in line with The International Centre for Diffraction Data (ICDD, United States card no. 080-0075). The well-defined peaks of the synthesized ZRTs structures are indicative of a single phase. The synthesized structures generated significant peaks that are suggestive of nanoscale range (Mohammadi and Ghasemi, 2018). No additional peaks signify that the sample is free from impurities. Moreover, the strong, narrow diffraction peaks depict the crystalline nature of the synthesized ZRT samples. The peaks in all XRD patterns, specifically [1 0 0], [0 0 2], [1 0 1], [1 0 2], [1 1 0], [1 0 3], [1 1 2] and [2 0 1] show diffraction peaks for ZnO nanostructures. This led to the identification of ZnO as an epitaxial phase with a hexagonal lattice (Umar et al., 2022). According to our previous study, the highest peak of 2θ occurred at 36.3°, which was reported all along the orientation [1 0 1] (Khan et al., 2016). Additionally, the peaks found along the orientation [0 0 2], [1 0 2], [1 1 0], and [1 0 3] indicated that ZnO has a pure wurtzite structure (Nilavukkarasi et al., 2020). This is in accordance with previous reports, and corroborates its purity (Nadia et al., 2019; Naseer et al., 2020).

The Debye-Scherrer-equation was used to determine the crystallite sizes (D) of the ZRTs from the peak with the highest intensity [1 0 1]. The sizes of the ZRT-1 and ZRT-2 specimens were estimated to be ∼800 nm and ∼667 nm, respectively. It was found that extending the time for introduction of ion carriers into the experimental setup resulted in smaller particles. Extending the period for ion carrier introduction into the experimental setup was found to have an adverse relationship with sample thickness, which affected the average diameter of ZRT specimens (Table 1).

**TABLE 1:** The effect of introduction time of ion-carriers (NaOH) on the synthesis of ZRTs: ZRT-1 and ZRT-2.

ZRT samples	Average nano-diameters (nm)	Average aspect ratio
ZRT-1	∼556 ± 1.7 nm	∼39.0 ± 0.13
ZRT-2	∼436 ± 2.3 nm	∼52.5 ± 0.21

The experimental design allowed for postponement of ion carrier entry, which facilitated uniform and better distribution with the capping agent, resulting in the expansion anywhere along longitudinal (c-axis) and a decrease in the width. This might be the reason behind the change in the optimal aspect ratios as the ion carriers were added more gradually into the experiment. The nano-diameters of the ZRT specimens are also reduced as a result. As one of the key factors of the size and shape, delay in the introduction of ion-carriers to experimental setup can lead to a different crystal formation. For ZRTs, it is determined that when NaOH is added, microtubule-like arrangement is the result of the difference in the crystal structures (Jung et al., 2008; Kumar et al., 2013).

#### 3.1.2 SEM analysis of ZRTs

**FIGURE 2:**
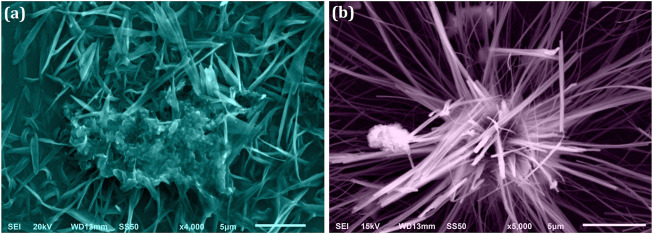
SEM image of ZRT samples prepared by varying reaction time of ion-carriers on the synthesis of zinc oxide nanoparticles (ZRTs): **(A)** ZRT-1 and **(B)** ZRT-2.

The microtubule-like arrangement for ZRTs samples was revealed in SEM photographs of ZRTs samples prepared at varying introduction time of ion-carriers in the production of ZRTs (Figure 2). SEM micrographs of ZRT-1 and ZRT-2 revealed microtubule like morphologies. Each cluster contains a good number of the hair strands arranged in hexagonal arrays. These fibril-like hexagonal arrays of ZRT-1 and ZRT-2 samples had average thickness of around ∼556 and ∼436 nm, respectively. This is in line with experimental XRD results (Figure 1) that demonstrates that the shrinking the ZRTs are a result of postponing the timeframe of ion carrier’s insertion in the reaction mixture. Furthermore, the optimal aspect ratios (L/D) ranged between ∼39.0 and ∼52.5, respectively, for ZRT specimens. The ZRT-1 SEM image reveals some agglomerated surface (Figure 2A) with no discernible shape and uniformly scattered nanoparticle (Bauermann et al., 2006). However, by delaying the ion carrier introduction, the ZRT-2 sample showed less extent of agglomeration (Figure 2B) (Khan et al, 2021).

We have noticed that the crystal size determined by XRD and the particle size calculated by SEM are very different. XRD determines the mean scattering domain size, known as the crystallite size which is distinct from the particle size determined by SEM. The loss of the secondary electrons (SE) (low-energy electrons belonging to sample) signals in SEM results in an edge effect. These SE might readily be protected by the particle’s up-and-down microstructure. Consequently, the edge that we perceive in a SEM image is occasionally not the particle’s actual boundary (Rao and Biswas 2009; Bandyopadhyay and Bose 2013). Since the crystallite “size” seen in SEM images is a two-dimensional cut through a three-dimensional structure; the observed “particle size” is not the real one, but a section through 3-dimensional crystal. Therefore, it is possible that the true crystal size as calculated by XRD is greater than the SEM image e.g. if the crystal presents its shortest dimensional axis (Staab et al., 1999).

#### 3.1.3 UV-visible spectroscopic analysis of ZRTs

**FIGURE 3:**
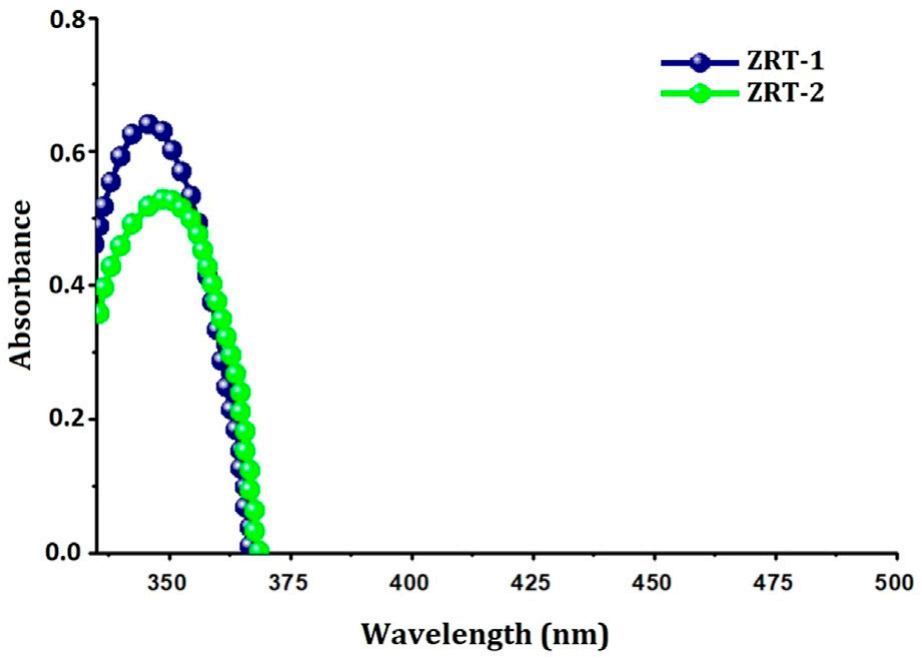
UV-Visible spectra of ZRT samples prepared by varying reaction time of ion-carriers on the synthesis of zinc oxide nanoparticles: ZRT-1 and ZRT-2.

To further elucidate the optical/structural properties, UV-Visible absorption spectroscopy was used (Figure 3). Both samples displayed significantly narrow absorption bands in the UV-A region between 364 and 382 nm devoid of any other peaks (Umar et al., 2022), signifying combined light wave and electron vibrations of nanoparticles (Miri et al., 2019). However, from ZRT-1 to ZRT-2, the absorption bands’ intensity enhanced slightly. The observed absorption peaks were typical of the wurtzite hexagonal structure (Davis et al., 2019). Additionally, it was discovered that altering the ion-carrier introduction time during the synthesis of ZRTs led to a slight change in the ZRTs’ absorption wavelength maxima (λ_max_) (Umar et al., 2022). This red-shift to longer wavelength may be due to the change in the aspect ratios, since the dimensions as well as morphologies of the ZRTs impacts the spectral properties (Basri et al., 2020; Kovács et al., 2022). As such, it has been reported that several characteristics, including crystal size, reaction temperature, the use of an ion carrier, the mechanism of synthesis used, the number of reacting molecules and pH (Kavitha and Kumar 2019; Basnet and Chatterjee 2020) affect the shape, size and spectral properties of the ZnO nanostructures (Mohammadi and Ghasemi, 2018).

Zinc oxide presents itself in three different crystal formations, viz wurtzite, zinc-blende, and the infrequently observed rock-salt (Ozgur et al., 2005; Moezzi et al., 2012). The lattice spacing of the hexagonal wurtzite structure is a = 0.325 nm and c = 0.521 nm, where c/a is 1.6, very close to the ideal value for a hexagonal cell (which is c/a = 1.633). In this arrangement, four oxygen atoms surround each tetrahedral Zn atom, and *vice–versa* (George et al., 2009). The structure is schematically shown as a number of consecutive layers of Zn and an O ion piled alongside the c-axis and is thermodynamically balanced in a micro-environment (Sirelkhatim et al., 2015). Zinc-blende structure is metastable and can be equilibrated *via* growth techniques.

### 3.2 Anticancer activity of ZRTs

Plenty of studies suggest that metal oxide nanoparticles can kill cancerous cells very specifically while sparing healthy cells (Vimala et al., 2017; Wang et al., 2020; Ahamed et al., 2021; Meng et al., 2022). Due to their unique physico-chemical characteristics, ZnO nanostructures in particular have demonstrated inherent selective cytotoxic action against a diverse range of cancer cells including ovarian (Bai et al., 2017; Alipour et al., 2022), lung (Rajeshkumar et al., 2018) lymphoma (Alsagaby, 2022), and laryngeal cancer (Wang et al., 2019). On the contrary, limited reports are available evaluating its anti-oncogenic potential towards epidermal cancer cells. In a recent investigation, it was discovered that ZRT prepared from aqueous extracts of *Cratoxylum formosum* leaves inhibited the viability of A431 cells in a dose-dependent manner while having no effect on healthy Vero cells (Jevapatarakul et al., 2020).

#### 3.2.1 Toxicity evaluation of ZRTs

**FIGURE 4:**
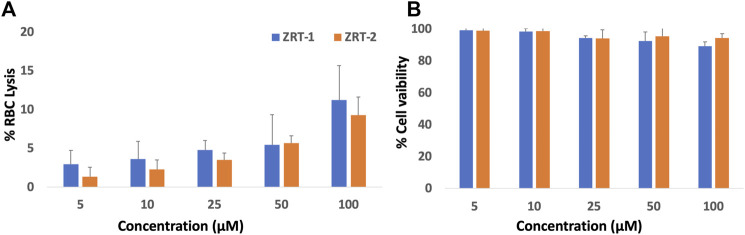
**(A)** Hemolytic activity of ZRTs nanoparticles: The extent of damage caused to red blood cells by the ZRT was measured as percent lysis of total erythrocytes used in the individual sample. **(B)**: *In vitro* cytotoxicity assay: Dose–response effects of ZRTs nanoparticles on cytotoxicity against Vero cells.

Toxicity evaluation is a must for every new formulation before introducing it into a clinical setting. Thus, we tested the intrinsic toxicity profile of ZRTs on healthy RBCs as well as normal mammalian Vero cell line prior to assessing its anti-cancer property. ZRTs showed no adverse effects on human RBC cells, even at high doses. Only 11.2 and 9.2% cell lysis were observed at a concentration as high as 100 μM of ZRT-1 and ZRT-2, respectively (Figure 4A). Further, both ZRT-1 and ZRT-2 displayed much lower toxicity to normal kidney Vero cells. Cell viability was reported to be 99.40%, 98.61%, 94.41%, 92.65%, and 89.32% at a concentration of 5, 50, and 100 μM respectively, for ZRT-1 (Figure 4B). In case of ZRT-2, the cell viability was reported to be 99.0%, 98.8%, 94.3%, 95.6%, 94.5% at the same concentration used. Hence, it can be concluded that the ZRTs are safe for healthy, normal cells. Considerable investigations have noted its non-toxic nature (Sirelkhatim et al., 2015; Bandala & Berli, 2019; Khan et al., 2020; Singh et al., 2020; Ahamed et al., 2021) supporting our previous study (Khan et al., 2021). Both *in vivo* tests on blood, normal tissues, and major organs, as well as *in vitro* evaluation of the toxicity of ZRTs on normal and undamaged human RBCs revealed no detrimental effects or toxicity (Vimala et al., 2017). Additionally, no evidence of geno-toxicity, carcinogenic effects, or reproductive toxicity in humans has been reported (Li et al., 2012; Siddiqi et al., 2018; Khan et al., 2020; Wojnarowicz et al., 2020). Moreover, investigations on ZRTs bio-distribution have found that even with high dosages (500 mg/kg), there is minimal damage to tissues (Wang et al., 2016). Thus, it can be concluded ZRTs can eradicate cancer cells with no or minimal harm to normal healthy mammalian cells (Wiesmann et al., 2019).

#### 3.2.2 Morphological alterations in cancer cells

**FIGURE 5:**
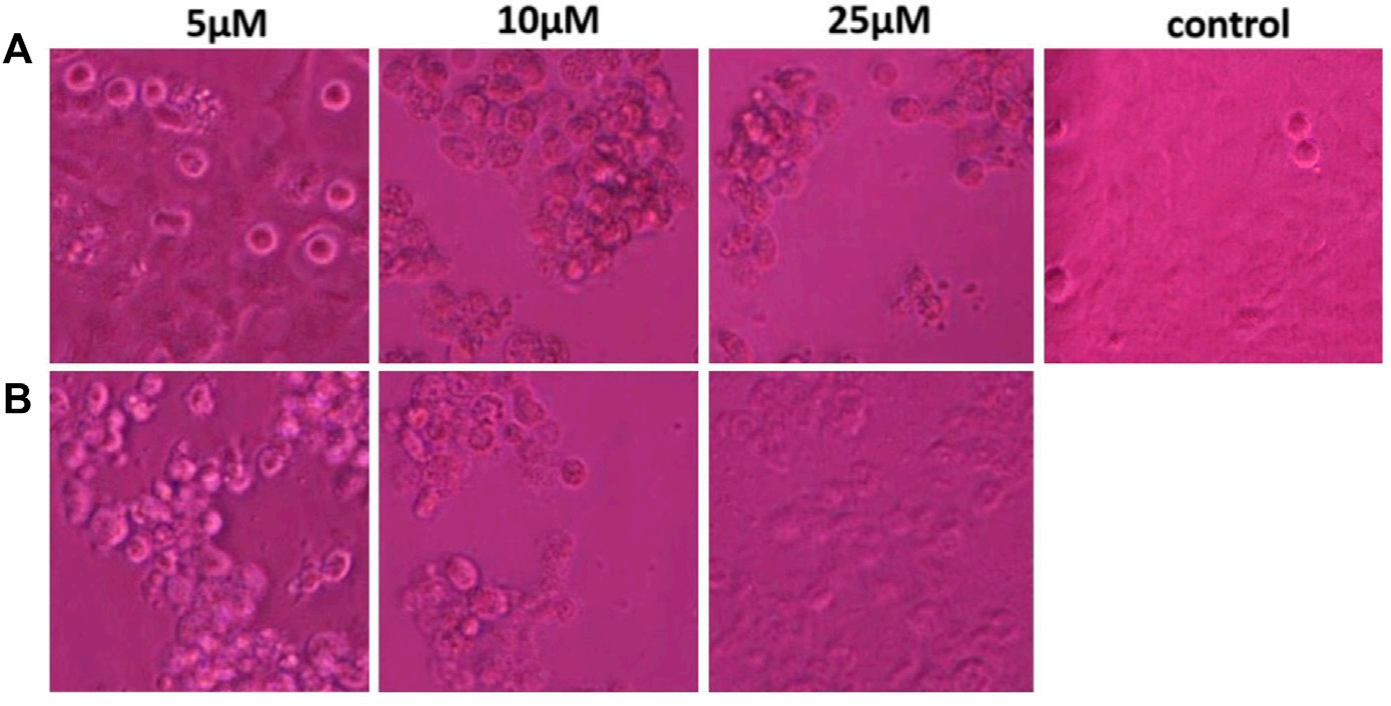
**(A,B)** Morphological view of live and dead cells of human epidermoid carcinoma A431 cell lines treated with control, 5 μM, 10 μM and 25 μM concentration of zinc oxide nano-particles, ZRT-1 and ZRT-2, respectively.

The anti-proliferative activity of ZRTs samples against the A431 epidermoid cancer cell lines at various dosages *viz.* 5, 10, 25 µM were observed (Figure 5). The untreated cells (control) maintained their smooth, flat, uniform cellular surface, indicating their healthy state. Contrastingly, the exposed cancer cells displayed typical apoptotic cell death as compared to the untreated cells. The A431 cells acquired a globular shape showing cellular shrinkage when treated with different ZRTs. Moreover, the number of cells with this morphology increased when concentration of ZRTs was raised. A431 cancer cells treated with ZRT-2 displayed a greater collection of rounded, shriveled cell arrangements than ZRT-1 treated cells (Figures 5A,B). Similar research has shown that ZRTs-treated cells undergo drastic morphological changes and form clusters in the media following their detachment from culture plates (Yadav et al., 2021). Our results revealed that ZRTs had a dose- and size-dependent effect on the morphology of the cancer cells. This is in accordance with previous studies that demonstrated that smaller particles displayed better cytotoxic activity better (Akter et al., 2018; Penders et al., 2017).

#### 3.2.3 MTT assay

**FIGURE 6:**
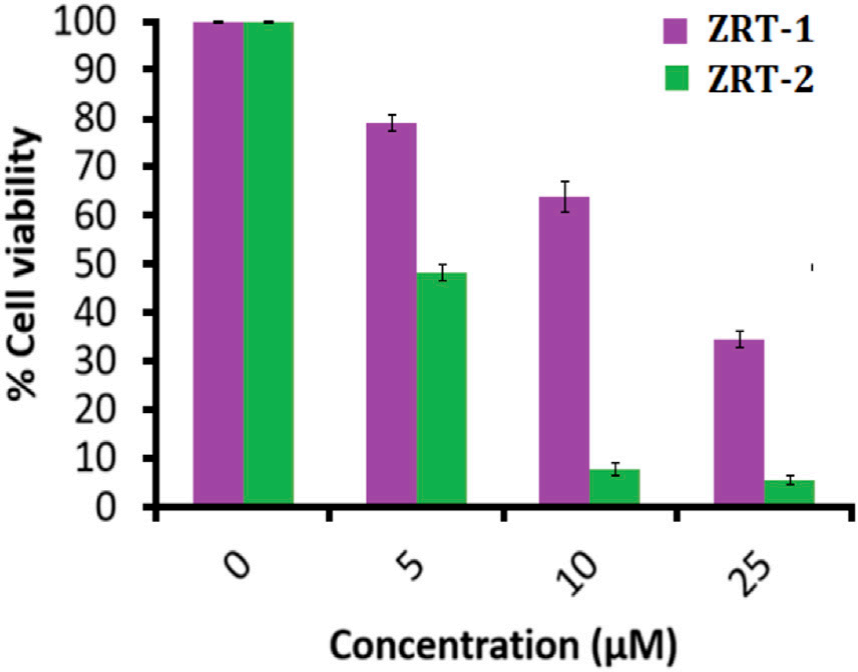
MTT assay: Percent cell viability of human epidermoid carcinoma A431 cells at 24 h. Values are expressed as mean ± SEM of at least three independent experiments, *p < 0.05 as compared with their respective control.

Meanwhile, additional MTT results showed that ZRT-2 significantly reduced viability of A431 cells in comparison to ZRT-1 (Figure 6). In accordance with the cytotoxic studies, ZRT-1 at 5 μM concentration lowered the vitality of the cells to about 78.98 ± 0.99% (*p* < 0.05) in comparison with the control. At 10 μM and 25 μM concentration of ZRT-1, the cell viability was significantly decreased to roughly 63.66 ± 1.88 and 34.48% ± 1.02% (*p* < 0.001), respectively. Likewise, 5 μM of ZRT-2 decreased the viability of the cells to about 48.32% ± 0.78% (*p* < 0.05) in comparison with the control. At 10 μM and 25 μM concentration, ZRT-2 decreased the cell viability to roughly 7.55 ± 0.27 and 5.56% ± 0.93% (*p* < 0.001), respectively. ZRT-1 and ZRT-2 were shown to have IC_50_ values of 8 μM and 6 μM against A431 cells, respectively. Additionally, IC_50_ values ZRTs have been reported in the literature to be roughly similar or even higher (Dobrucka et al., 2018, Dobrucka et al., 2020).

#### 3.2.4 Effect of ZRTs on intracellular ROS generation

**FIGURE 7:**
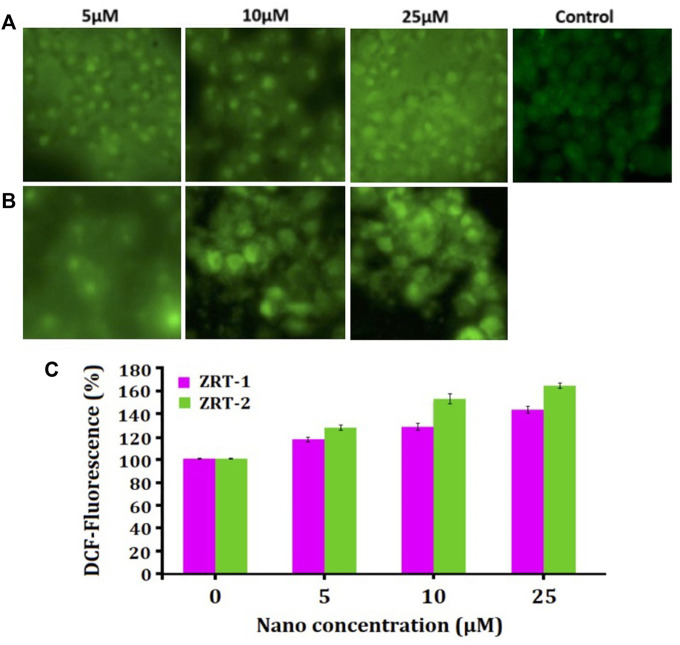
**(A,B)**: Photomicrographs showing intra-cellular ROS generation in human epidermoid carcinoma A431 cell lines induced by control, 5 μM, 10 μM, and 25 μM concentration of zinc oxide nano-particles, ZRT-1 and ZRT-2, respectively after 12 h incubation and stained with DCFH-DA. **(C)** Graph showing extent of ROS generation expressed as the percentage of fluorescence intensity relative to the control. Values are expressed as mean ± SEM of at least three independent experiments, *p < 0.05 as compared with their respective control.

ZRTs substantially increased ROS amount in A431 cells in a dose-sensitive manner in comparison with the control (Figure 7). Quantitative analysis of ROS disclosed that 5 μM of ZRT-1 increased ROS generation by 117.24% (*p* < 0.01) (Figure 7A), while ZRT-2 at the same concentration enhanced ROS level by 129.28% (*p* < 0.01) (Figure 7B). Furthermore, ROS levels were elevated by 130.00 and 143.32% (*p* < 0.001) than control at 10 and 25 μM concentration of ZRT-1, respectively. Likewise, similar concentration of ZRT-2 led to the increase in ROS production by 152.82 and 166.71% (*p* < 0.001) respectively, against control (Figure 7C).

#### 3.2.5 Effect of ZRTs on chromatin condensation

**FIGURE 8:**
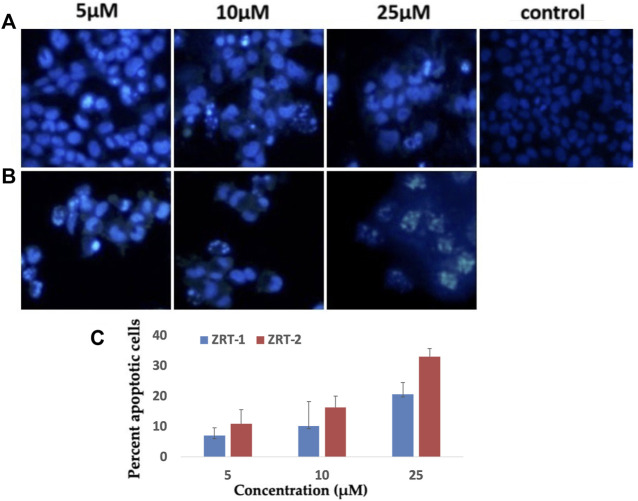
**(A,B)**: Human epidermoid carcinoma A431 cell lines were treated with control, 5 μM, 10 μM and 25 μM concentration of zinc oxide nano-particles, ZRT-1 and ZRT-2, respectively and stained with DAPI **(C)** Representative graphs showing the numerical data of the percent apoptotic cells against the concentration of zinc oxide nano-particles, ZRT-1 and ZRT-2. Values are expressed as mean ± SEM of at least three independent experiments, *p < 0.05 as compared with their respective control.

Upon treatment with 10 μM of ZRTs, the chromatin condensation inside A431 cells increased significantly when compared with the control cells. Also, maximum condensation was seen at 25 μM concentration of ZRTs (Figures 8A,B). With ZRT-1, 10.2% and 20.67% apoptotic cells were observed; while, approximately 16.3% and 33.0% of apoptotic cells were observed for ZRT-2 at 10 and 25 μM concentration, respectively (Figure 8C). The abridged nuclei in A431 cells are suggestive of cell killing by apoptosis.

Although the exact mechanism(s) underlying the cytotoxicity of ZRTs are still being investigated, it is widely accepted that the generation of reactive oxygen species (ROS) is a significant contributing component. Thus, it can speculated that the primary cause of ZRTs’ cytotoxicity for cancer cells is their unique capability to cause oxidative stress in cancer cells. This typical feature stems from its semiconductor behavior. When the antioxidant capacity of the target cell is surpassed, ZRTs increase the production of ROS, which causes oxidative stress and ultimately cell death (Bisht and Rayamajhi, 2016).

Broadly speaking, moderate quantities of ROS are required for essential cellular functions, such as cellular growth and differentiation; nonetheless, vast amounts of ROS constitute a serious hazard that may ultimately result in DNA damage leading to untimely induction of programmed cell death (PCD) (Huang et al., 2019). The diverse pathways by which carcinoma cells perish in response to elevated levels of ROS seriously damages the protein, DNA, and RNA components in the process (Ott et al., 2007; Kao et al., 2017). It was found that ZRT-2 generated a little bit more ROS than ZRT-1, suggesting that ZRT-2 was slightly more efficient at causing oxidative stress to kill A431 cells. Previous researchees have demonstrated a connection between the emergence of oxidative stress in several cancer cell lines including Hep-2, A549, BEAS-2B and lung cancer cells and the killing nature of diverse nanostructures (Manke et al., 2013).

## 4 Conclusion

This work discusses the fabrication of ZRTs by altering the entry time of ion-carriers to the experimental setup. XRD results confirmed the wurtzite crystalline nature of ZRTs, while SEM and UV-visible spectroscopy illustrated size and shape differences between ZRT-1 and ZRT-2. A shift in spectrum behavior was also seen when the ion-carriers’ introduction time was prolonged. This also led to changes in anti-proliferative behavior of ZRT specimens. Our finding was that ZRT-2 had a more severe impact against the human epidermoid cancer cells as compared to ZRT-1. ZRT-2 samples exhibited elevated ROS production and enhanced nuclear condensation, which in turn caused cell death and nuclear apoptosis. Our study opens new vistas for the application of ZRTs as chemotherapeutic drugs. Further *in vivo* studies should be performed to ascertain its full anticancer potential.

## Data Availability

The original contributions presented in the study are included in the article/Supplementary Material, further inquiries can be directed to the corresponding authors.
